# Splenic Vein Thrombosis: A Case Series of Consequential Chronic Pancreatitis and Sequential Myeloproliferative Disorder

**DOI:** 10.7759/cureus.25924

**Published:** 2022-06-14

**Authors:** Mansoor Zafar, William Heslop-Harrison, Linda Loterh, Kofi Ofuafor

**Affiliations:** 1 Gastroenterology, Hepatobiliary, and Hepatology, Royal Sussex County Hospital, University Hospitals Sussex NHS Foundation Trust, Brighton, GBR; 2 General Internal Medicine and Gastroenterology, Royal Sussex County Hospital, University Hospitals Sussex NHS Foundation Trust, Brighton, GBR; 3 Gastroenterology, Royal Sussex County Hospital, University Hospitals Sussex NHS Foundation Trust, Brighton, GBR

**Keywords:** polycythemia rubra vera, direct-acting oral anticoagulants, myeloproliferative disorder, chronic pancreatitis, splenic vein thrombosis

## Abstract

We present a case series of two patients with splenic vein thrombosis (SVT), a relatively uncommon condition supposed to occur in the context of pancreatitis or pancreatic malignancies. Splenic vein thrombosis may also be seen in cases of chronic pancreatitis, as in one of our patients. At times, splenic vein thrombosis may present with an incidental, isolated finding of gastric varices on computed tomography pulmonary angiogram (CTPA) while investigating for pulmonary embolus; such a result should prompt further investigation to rule out associated splenic vein thrombosis in a sequence. We attempt to highlight the importance of performing a blood count and hematocrit, supplementary to a liver screen, in another patient where the cause was not related to liver disease, but to myeloproliferative disorder, which resulted in a hyper-thrombotic state and splenic vein thrombosis as a consequence.

## Introduction

The deoxygenated blood from the abdomen returns to the right heart via two circulation systems, which include two venous channels: the first via the inferior vena cava to the right atrium and the second via the portal circulation. Together, this is labeled as the splanchnic circulation. Thrombosis of the splanchnic circulation could be due to various pathological etiologies involving all or subsections of this circulation. This includes mesenteric, hepatic, celiac, superior mesenteric, inferior mesenteric, portal, or splenic vein thrombosis (SVT) [1]. The incidence and prevalence of splanchnic vein thrombosis have been reported to have risen from 1970 to 2000 [2]. This is proposed to be related to increased access to computed tomography (CT) [2]. The most important cause of portal vein thrombosis has been reported to be cirrhosis. Autopsy findings have suggested the prevalence in cirrhotic patients to range from 6% to 64%. The prevalence in non-cirrhotic patients remains unknown [3]. Chronic pancreatitis has been reported to be the most common cause of splenic vein thrombosis (SVT) with prevalence reported to be between 5% and 22% [4]. The incidence of hepatic vein thrombosis (Budd-Chiari syndrome) has been reported to be 2.2 per million among females and two per million among males [5]. The incidence of mesenteric thrombosis leading to mesenteric ischemia has been reported to be between 2% and 10% of cases [6,7]. Lastly, the prevalence of SVT with underlying myeloproliferative disorder has been reported to be between 5% and 70%, signifying the importance of complete early investigation for prompt management [8].

## Case presentation

Case 1

A 64-year-old male with a background of pancreatic divisum, stable pseudocyst and chronic pancreatitis (two years), hiatus hernia, and pernicious anemia presented to the emergency department (ED) with sudden-onset right-sided pleuritic chest pain. His observations were stable, and on clinical examination, no abnormality was detected. ECG showed a sinus rhythm of 87 beats/minute with a right bundle branch block. His calculated Wells’ score based on history, presentation, and blood test results for pulmonary embolism (PE) was only 3 (moderate-risk group with a risk of PE of 16%). However, a raised D-dimer, right bundle branch block, and strong clinical suspicion for PE prompted a request for urgent computed tomography pulmonary angiogram (CTPA) (Table 1).

**Table 1:** Blood test results and calculated Wells’ score of 3. Source: Laboratory at Royal Sussex County Hospital, UK

Laboratory parameter	Unit	Range	Test result
Hemoglobin (Hb)	g/L	135-180	161
Serum C-reactive protein (CRP)	mg/L	0-5	169
White blood cell (WBC) count	× 10^9^/L	4-10	7.3
Neutrophils	× 10^9^/L	2-7	4.7
Platelets	× 10^9^/L	150-410	151
Basophils	× 10^9^/L	0-0.1	0.1
Monocytes	× 10^9^/L	0.2-1	0.6
Lymphocytes	× 10^9^/L	1-3	1.3
International normalization ratio (INR)	-	0.8-1.2	1.5
Serum troponin T	ng/L	0-14	5.70
D-dimer assay	ug/mL	0-0.64	4.53
Fibrinogen	g/L	1.8-4.5	3.2
Serum NT-proBNP	pg/mL	0-210	277
Serum urea	mmol/L	2.8-8.1	2
Serum creatinine	umol/L	62-106	52
Effective glomerular filtration rate (GFR) per 1.73 square meters	mL/minute	60-90	>60
Calculated Wells’ score	-	0	3

**Figure 1:**
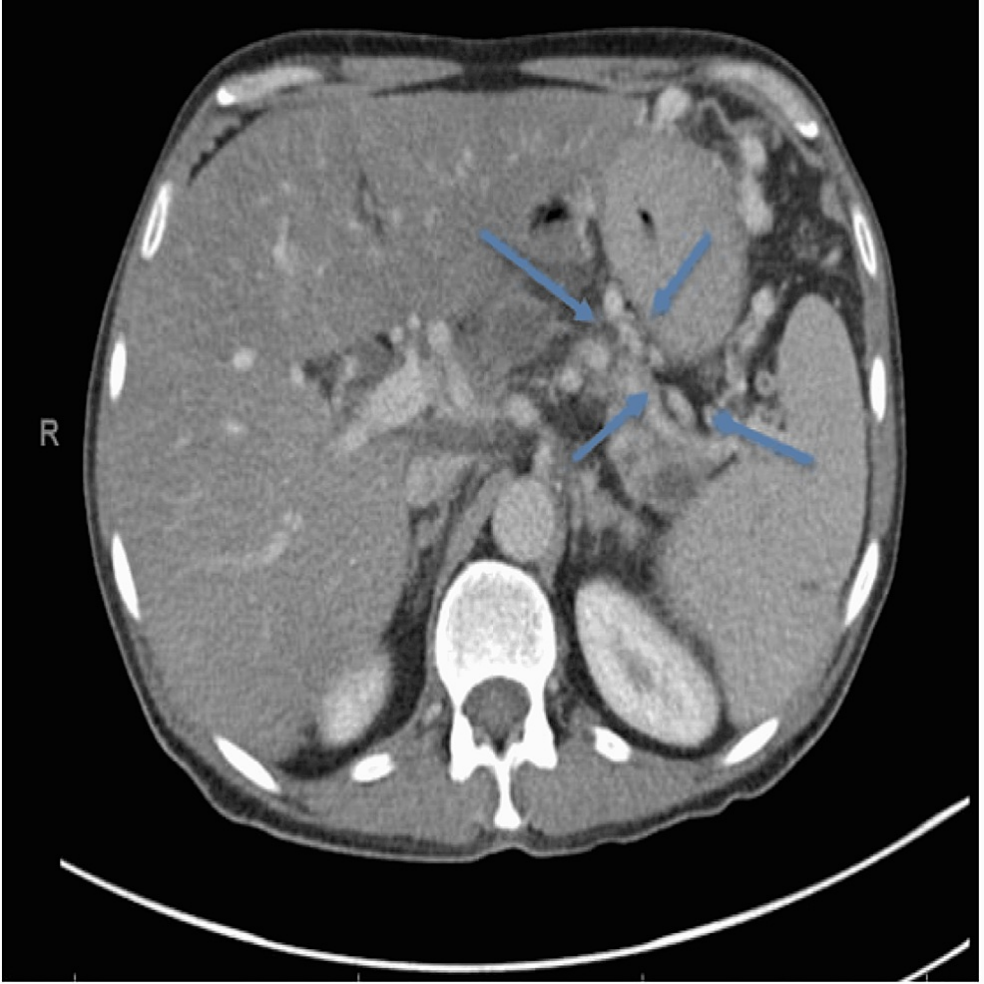
Computed tomography pulmonary angiogram (CTPA) with gastric varices (blue arrows).

This demonstrated right upper and lower lobe pulmonary emboli with further emboli in nearly all segmental arteries, along with a dilated right ventricle in keeping with right heart strain. The CTPA findings also reported a query regarding peri-gastric varices (Figure 1).

**Figure 2:**
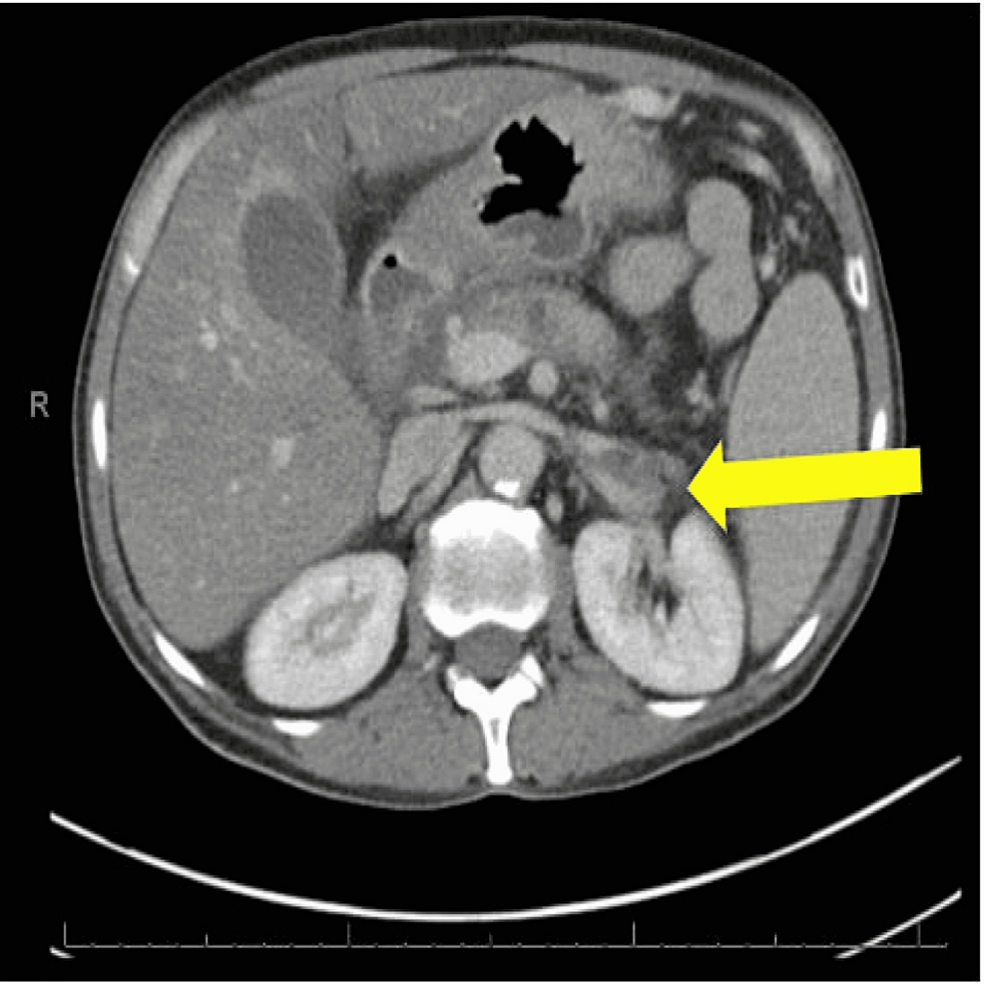
Computed tomography (cross-sectional view) with splenic vein thrombosis (yellow arrow).

At this time, a decision was made to request a CT of the abdomen and pelvis to rule out any splenic vein thrombosis associated with peri-gastric varices. The CT of the abdomen and pelvis showed evidence of fatty liver, left-sided gastric varices, and proximally occluded splenic vein with atheroma in the superior mesenteric artery (Figure 2).

**Figure 3:**
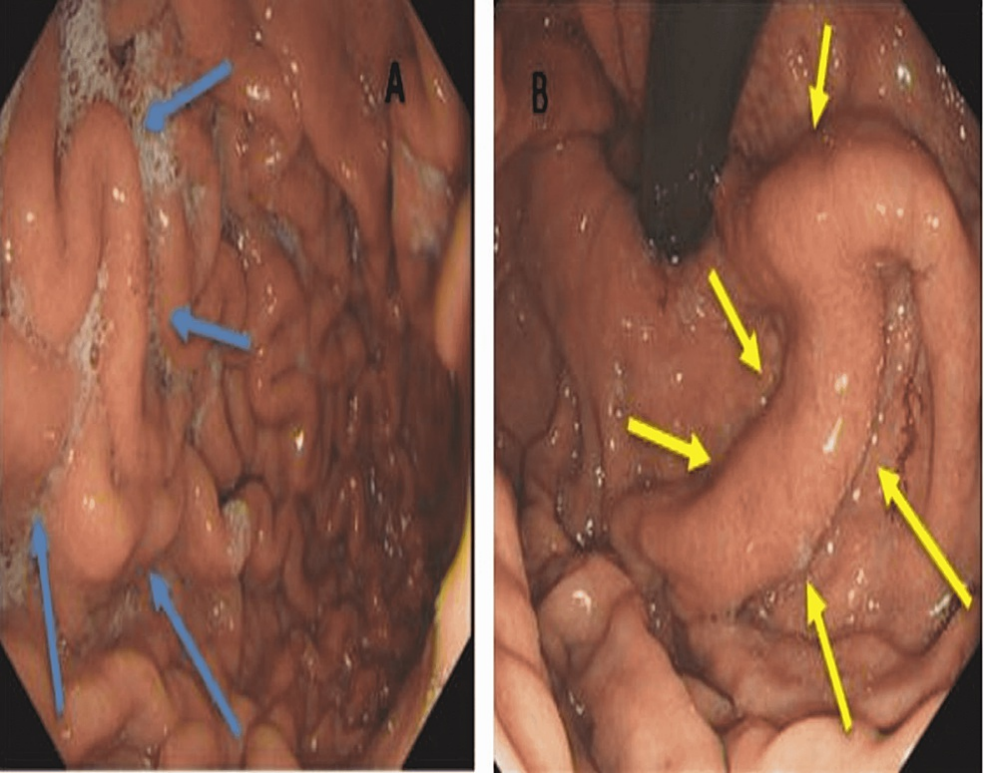
Upper orogastroduodenoscopy. A) Prominent gastric fold along the proximal stomach (blue arrows). B) Stomach in retroflexion with a prominent gastric fold along the proximal stomach (yellow arrows).

Additional findings were cystic masses around the tail of the pancreas along with calcific foci in the pancreas, in line with the previous history of chronic stable pancreatitis. A diagnosis of splenic vein thrombosis was confirmed along with left-sided gastric varices. The patient was started on a heparin infusion, while a request was submitted for upper orogastroduodenoscopy (OGD). The OGD did not demonstrate any gastric varices; however, a prominent gastric fold along the proximal stomach was seen (Figure 3). After 24 hours, the patient was switched from the heparin infusion to apixaban 5 mg twice a day. He was discharged with follow-up in the anticoagulation clinic.

Case 2

**Figure 4:**
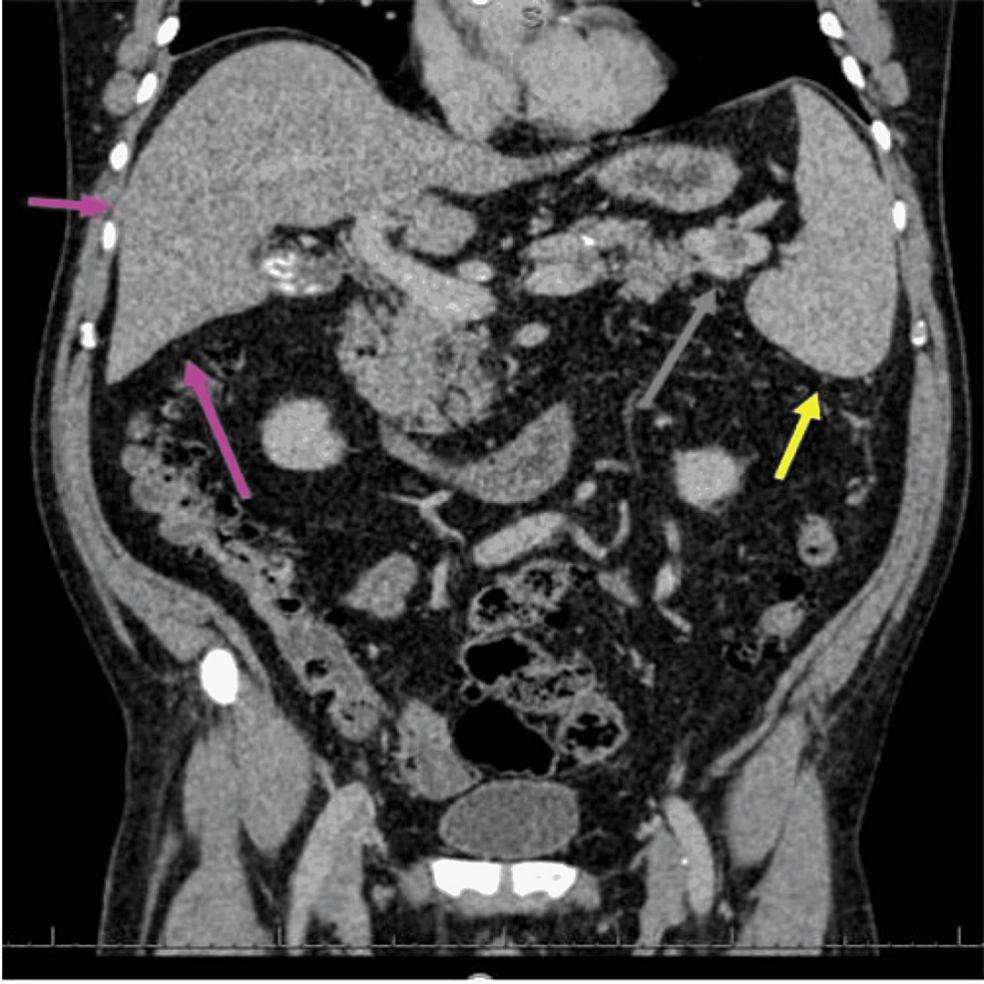
Computed tomography (sagittal view) with the irregular contour of the liver (pink arrows), partially occluded splenic vein thrombus (blue arrow), and splenomegaly (yellow arrow).

A 71-year-old male presented to the ED with a one-day history of left upper abdominal pain, reported 9 out of 10 severity. He had no significant past medical history, maintained an active lifestyle, did not smoke, and reported consuming between 8 and 10 units of alcohol per week. Clinical examination showed tenderness along the left hypochondrium along with a palpable spleen. There was no guarding, rigidity, or shifting dullness, with normal bowel sounds. Chest examination was unremarkable. While awaiting initial blood test results, an urgent CT of the abdomen and pelvis demonstrated a partially occluded SVT, splenomegaly of 18 cm, and irregular contour of the liver with an impression of cirrhosis (Figure 4).

His blood tests for liver function were deranged with raised alkaline phosphatase (ALP) and a mild rise in alanine transaminase (ALT) with normal bilirubin. However, the elevated hemoglobin, hematocrit, and white blood cell count and a high platelet count along with low erythropoietin level were suspicious for an underlying myeloproliferative disorder (Table 2). Later, he was found to be positive for the JAK2 mutation, with an otherwise normal myeloma screen.

**Table 2:** Blood test results with low erythropoietin level. Source: Laboratory at Royal Sussex County Hospital, UK

Laboratory parameter	Unit	Range	Test result
Hemoglobin (Hb)	g/L	83-101	179
White blood cell (WBC) count	× 10^9^/L	4-10	14.3
Platelets	× 10^9^/L	150-410	795
Red blood cell (RBC) count	× 10^12^/L	4.5-5.5	5.73
Hematocrit (HCT)	-	0.4-0.54	0.562
Mean corpuscular volume (MCV)	fL	20	98.1
Alanine transaminase (ALT)	IU/L	0-41	85
Alkaline phosphatase (ALP)	IU/L	40-129	235
Bilirubin	umol/L	0-21	16
Serum erythropoietin level	IU/L	4.3-29	<2.5

**Figure 5:**
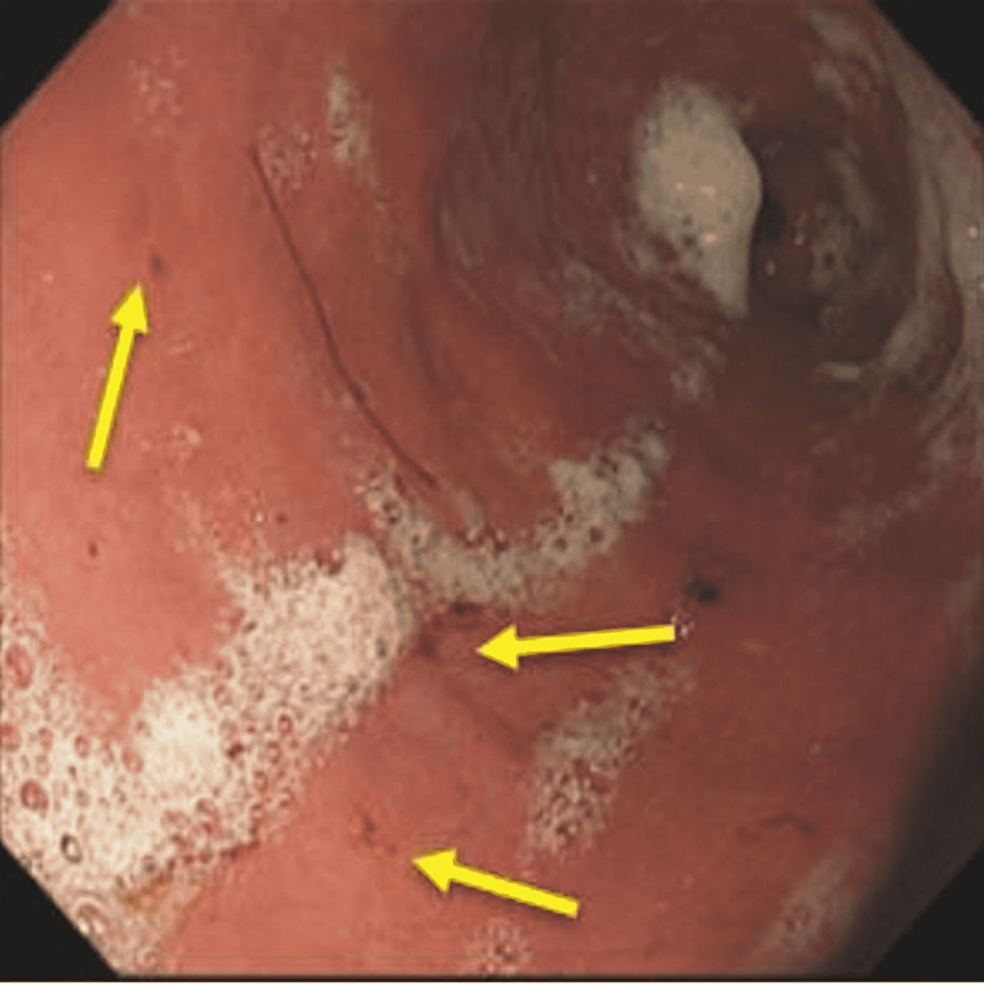
Orogastroduodenoscopy (OGD) demonstrating no evidence of varices, no portal gastropathy, and mild gastritis (yellow arrows).

A noninvasive liver screen was performed, including hepatitis screening, antibody profile, immunoglobulin, and fasting lipid profile, under the care of the gastroenterology team; these were inconclusive. He was started on subcutaneous enoxaparin of 1.5 mg/kg body weight to reduce the risk of future thrombotic events. He underwent an OGD, which showed no evidence of varices and mild gastritis (Figure 5).

A diagnosis of polycythemia rubra vera (PRV), a form of myeloproliferative disorder, was made in consultation with the hematology team. He was discharged home after switching from enoxaparin subcutaneous injections to oral rivaroxaban 20 mg once a day (OD), with hydroxycarbamide to suppress the platelet count, and omeprazole 20 mg OD. He was also booked for periodic venesection of 45 mL under the care of the hematology team with the aim to reduce hematocrit to less than 0.45. Ongoing follow-up in the hepatology clinic for periodic surveillance ultrasound was arranged.

## Discussion

Patients with SVT may present with abdominal pain with or without splenomegaly, thrombocytopenia, or variceal bleeding [9]. Paradoxically, if gastric varices are seen or queried, there is a need to rule out SVT [10]. Unlike patients with chronic liver disease with deranged LFTs, the LFTs are usually within the normal range in patients with SVT [11]. Enlarged retroperitoneal lymph nodes and pancreatic or peri-splenic nodes have been reported to be associated with inciting SVT [12]. However, recent data suggests acute or chronic pancreatitis in the pancreatic tail to be the inciting factor in most cases of SVT [13,14].

Vitamin B12 deficiency is known to be associated with increased homocysteine levels (hyperhomocysteinemia); vitamin B12 is needed as a cofactor to remethylate homocysteine to methionine. This hyperhomocysteinemia, reported to be associated with neurological symptoms arising from vitamin B12 deficiency (and with nitrous oxide abuse) [15], can increase the propensity for arterial and venous thrombosis [16] and therefore has also been reported to be associated with SVT [17].

Ultrasound of the abdomen with or without Doppler and CT have all been successfully used to diagnose SVT [18]. The need for imaging is most important during the initial stages when clinical examination findings may be unremarkable [18]. This suggests that earlier imaging would assist in obtaining a prompt diagnosis and management plan, facilitating patient discharge from the hospital, and reducing the burden on both hospital resources and patient emotional well-being.

Although the deteriorating patient can be managed with transcatheter thrombolysis or a transjugular intrahepatic portosystemic shunt (TIPS) [18], generally, stable patients who are diagnosed in the early stages can be managed with direct oral anticoagulants (DOACs). Subcutaneous injections of enoxaparin are also being used, mainly as bridging therapy. Patients with decompensated chronic liver disease are usually managed with warfarin [19].

## Conclusions

It is important to remember that splenic vein thrombosis has various causes, and although it may be more commonly associated with pancreatitis or pancreatic malignancy, it may also occur in association with myeloproliferative disorders. Although vitamin B 12 deficiency (with or without pernicious anemia) and/or nitrous oxide abuse are more commonly associated with neurological complications, splenic or splanchnic thrombosis should not be overlooked. Paradoxically, when identifying isolated gastric varices, it is important to maintain a high degree of suspicion for possible associated splenic vein thrombosis.

A prompt diagnosis followed by appropriate management with anticoagulants not only helps avoid complications but also reduces hospital costs and emotional burden for patients and their relatives and facilitates early discharge, allowing patients to be managed in the community setting.
